# Viral Small Interfering RNAs Target Host Genes to Mediate Disease Symptoms in Plants

**DOI:** 10.1371/journal.ppat.1002022

**Published:** 2011-05-05

**Authors:** Neil A. Smith, Andrew L. Eamens, Ming-Bo Wang

**Affiliations:** CSIRO Plant Industry, Canberra, Australia; University of California Riverside, United States of America

## Abstract

The *Cucumber mosaic virus* (CMV) Y-satellite RNA (Y-Sat) has a small non-protein-coding RNA genome that induces yellowing symptoms in infected *Nicotiana tabacum* (tobacco). How this RNA pathogen induces such symptoms has been a longstanding question. We show that the yellowing symptoms are a result of small interfering RNA (siRNA)-directed RNA silencing of the chlorophyll biosynthetic gene, CHLI. The CHLI mRNA contains a 22-nucleotide (nt) complementary sequence to the Y-Sat genome, and in Y-Sat-infected plants, CHLI expression is dramatically down-regulated. Small RNA sequencing and 5′ RACE analyses confirmed that this 22-nt sequence was targeted for mRNA cleavage by Y-Sat-derived siRNAs. Transformation of tobacco with a RNA interference (RNAi) vector targeting CHLI induced Y-Sat-like symptoms. In addition, the symptoms of Y-Sat infection can be completely prevented by transforming tobacco with a silencing-resistant variant of the CHLI gene. These results suggest that siRNA-directed silencing of CHLI is solely responsible for the Y-Sat-induced symptoms. Furthermore, we demonstrate that two *Nicotiana* species, which do not develop yellowing symptoms upon Y-Sat infection, contain a single nucleotide polymorphism within the siRNA-targeted CHLI sequence. This suggests that the previously observed species specificity of Y-Sat-induced symptoms is due to natural sequence variation in the CHLI gene, preventing CHLI silencing in species with a mismatch to the Y-Sat siRNA. Taken together, these findings provide the first demonstration of small RNA-mediated viral disease symptom production and offer an explanation of the species specificity of the viral disease.

## Author Summary

Viral infections result in a variety of disease symptoms that vary in character and severity depending on the type of viral infection and individual host factors. Despite extensive research, the molecular basis of viral disease development has remained poorly understood. Both plant and animal viruses express 20–25 nucleotide siRNAs or microRNAs (miRNAs) in their host. These virus-specific small RNAs (sRNAs) direct RNA silencing of homologous viral genes to restrict virus replication forming part of the host's antiviral defense mechanism. Using a plant viral satellite RNA as a model system, we demonstrate here a new function for virus-derived sRNAs: induction of disease symptoms by silencing of a physiologically important host gene. Furthermore, we demonstrate that such viral-derived sRNA-induced disease symptoms can be prevented by the expression of a either naturally evolved, or artificially introduced, silencing-resistant sequence variant of the viral siRNA-targeted gene. These findings not only provide the first demonstration of a sRNA-mediated viral disease mechanism, but also offer an alternate strategy to prevent the onset of such viral diseases.

## Introduction

Plant viruses are often accompanied by small parasitic RNAs termed satellite RNAs. Satellite RNAs range in size from ∼220 to 1400 nucleotides (nt) in length and depend on their associated viruses (known as the helper virus) for replication, encapsidation, movement and transmission, but share little or no sequence homology to the helper virus itself [1]. Most satellite RNAs do not encode functional proteins, yet can induce disease symptoms which range from chlorosis and necrosis, to total death of the infected plant [1], [2]. How such non-protein-coding RNA pathogens induce disease symptoms has been a longstanding question. Early studies showed that the pathogenicity of a satellite RNA is determined at the nucleotide level, with one to several nucleotide changes dramatically altering both the virulence and host specificity of disease induction [2]–[6]. Subsequent studies demonstrated that satellite RNA replication is associated with the accumulation of high levels of satellite RNA-derived small interfering RNAs (siRNA) [7]. This class of small RNA (sRNA) has been shown to direct RNA silencing in plants through sequence-specific mRNA cleavage or DNA methylation [8], [9]. Taken together, these findings led to the suggestion that pathogenic satellite-derived siRNAs might have sequence complementarity to a physiologically important host gene, and that the observed disease symptoms are in fact due to satellite siRNA-directed silencing of the targeted host gene [10]. However, to date, no such host gene has been identified, leaving the satellite RNA-induced disease mechanism unsolved. In this report we explore the sRNA-mediated disease mechanism using the Y-satellite of *Cucumber mosaic virus* (CMV Y-Sat). The CMV Y-Sat consists of a 369-nt single-stranded RNA genome and induces distinct yellowing symptoms in a number of *Nicotiana* species including *N. tabacum* (tobacco) [11]. We show that the Y-Sat-induced yellowing symptoms result from Y-Sat siRNA-directed silencing of the host chlorophyll biosynthetic gene, CHLI. Furthermore, we demonstrate that Y-Sat-induced symptoms can be prevented by transforming tobacco with a silencing-resistant version of CHLI and provide evidence that the observed species specificity of Y-Sat-induced disease symptoms is due to natural sequence variation within the targeted region of the CHLI transcript.

## Results

### The chlorophyll biosynthetic gene CHLI is silenced by Y-Sat-derived siRNAs

**Figure 1:**
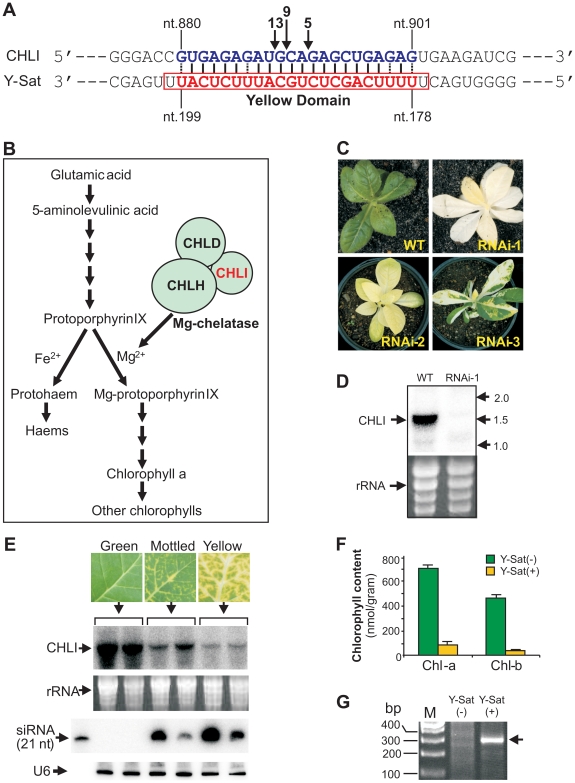
The tobacco chlorophyll biosynthetic pathway gene CHLI is silenced upon CMV Y-Sat infection. (***A***), The Y-Sat yellow domain matches a 22-nt sequence in the CHLI coding region. (***B***), CHLI is a key component in the chlorophyll biosynthesis pathway. (***C***) and (***D***), *N. tabacum* plants transformed with a CHLI RNAi construct (CHLI-RNAi; Figure 3*B*) show leaf yellowing (*C*) that is associated with dramatic down-regulation of the CHLI gene (*D*). (***E***), The CHLI gene is silenced in CMV Y-Sat-infected tobacco plants. The top panel shows the analysed tobacco leaf tissues with different levels of yellowing symptoms. The middle two panels are of a northern blot gel showing down-regulation of CHLI mRNA upon CMV Y-Sat infection. The bottom two panels are of a small RNA northern blot gel hybridized with a 21-nt Locked Nucleic Acids (LNA) probe (5′ ATGAGAAATGCAGAGCTGAAA 3′) complementary to the CHLI-targeting Y-Sat siRNA (from nt. 179). Note that the severity of the yellowing symptoms correlates with the degree of CHLI silencing, which in turn shows good correlation with the level of Y-Sat siRNAs. (***F***), The content of two major chlorophylls (Chl-a and Chl-b) is dramatically reduced in CMV Y-Sat-infected tobacco leaves. (***G***), A unique CHLI fragment is amplified by 5′ RACE from the CMV Y-Sat-infected tobacco but not from the uninfected tissue, indicating Y-Sat siRNA-directed cleavage of the CHLI transcript at the predicted target site. The exact cleavage sites are indicated by arrows in (*1A*), and the number of sequenced clones for the respective cleavage product is given above each arrow.

The nucleotide sequence responsible for the yellowing symptoms of the CMV satellite disease has been mapped to a small 24-nt region of the Y-Sat genome (nt. 177–200 and Figure 1*A*, referred to hereon as the ‘yellow domain’) [11]–[14]. Genetic analysis of progeny plants from a cross between the disease-susceptible species *N. bigelovii* and the disease-resistant species *N. clevelandii*, suggested that the yellowing symptoms induced upon CMV Y-Sat infection are associated with a single, incompletely dominant gene in the *Nicotiana* species [15]. We hypothesized that a siRNA derived from the yellow domain was directing RNA silencing of a tobacco gene, which in turn led to the expression of the observed yellowing symptoms. BLAST searches with the 24-nt yellow domain sequence against tobacco sequences in the NCBI database were performed to identify 21-nt or longer target sequences. No cDNA with perfect 21-nt complementarity was identified, however, accommodating weak G:U base pairings as a match allowed for the identification of a single *N. tabacum* sequence complementary to 22 nt of the Y-Sat yellow domain (from nt. 178 to nt.199; Figure 1*A*). The identified sequence is part of the coding region of the magnesium chelatase subunit CHLI, a 426-amino acid protein essential for chlorophyll biosynthesis (Figure 1*B*; see Text S1 for sequences). Previous studies have shown that Y-Sat-induced yellowing symptoms are associated with reduced chlorophyll content [16], making CHLI a strong target candidate for Y-Sat siRNA-directed silencing. Transformation of *N. tabacum* with a RNA interference (RNAi) vector targeting CHLI resulted in a dramatic decrease in CHLI expression and severe chlorosis of the transgenic plants, which ranged from yellowing to complete bleaching (Figure 1*C*–*D*). The phenotypes expressed by RNAi plants parallels the appearance of Y-Sat-induced symptoms, and are consistent with CHLI silencing being responsible for the disease phenotype.

Northern blot analysis confirmed that CHLI mRNA was dramatically down-regulated upon CMV Y-Sat infection (Figure 1*E*). CHLI expression was not affected by infection with the CMV helper virus alone (Figure S1). The level of CHLI down-regulation in CMV Y-Sat-infected plants correlated strongly with the severity of chlorosis, and with the accumulation of yellow domain-specific siRNAs (Figure 1*E*). As expected, CHLI down-regulation was associated with a dramatic reduction in chlorophyll content (Figure 1*F*). 5′ rapid amplification of cDNA ends (5′ RACE) on RNA samples extracted from CMV Y-Sat-infected and uninfected tobacco plants showed that the down-regulation of CHLI was due to siRNA-directed RNA cleavage at the predicted 22-nt target site. An expected 310-base pair (bp) product was amplified from the CMV Y-Sat-infected plants; however, no such product could be amplified from RNA extracts of uninfected tobacco (Figure 1*G*). Sequencing of the 5′ RACE product showed that RNA cleavage occurred at three distinct positions within the 22-nt Y-Sat siRNA-targeted CHLI sequence (Figure 1*A*).

**Figure 2:**
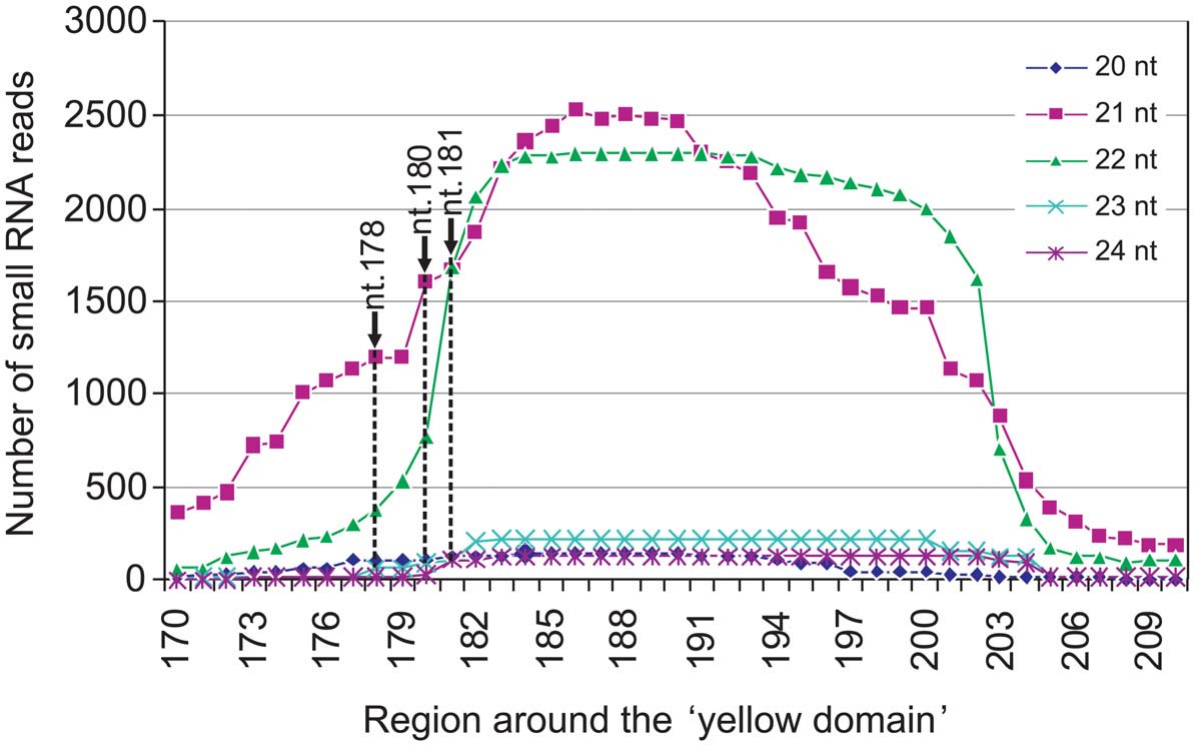
Y-Sat siRNA distribution around the yellow domain of the CMV Y-Sat genome detected by small RNA deep sequencing. Note that most siRNAs are of 21 and 22-nt size classes, which are effective in directing mRNA cleavage [8], [9]. Each point represents the number of reads (the Y-axis), and the position of the 5′ terminal nucleotide along the Y-Sat genome (the X-axis), of a specific siRNA. The three dashed lines indicate the 5′ terminal nucleotide of the three siRNAs that can direct CHLI cleavage at the sites as determined by 5′ RACE.

siRNA-directed cleavage of a target RNA normally occurs across nucleotides 10 and 11 of the siRNA [17]. The three cleavage sites detected by the 5′ RACE analyses therefore implied that the silencing of CHLI is directed by three individual Y-Sat siRNA species with their 5′-terminal nucleotides corresponding to nt. 178, 180 and 181 of the Y-Sat RNA genome respectively (Figure 1*A*). To confirm that these specific siRNAs were present, the total sRNA population of CMV Y-Sat-infected tobacco plants was sequenced using Solexa technology. Approximately 4 million sRNA reads were obtained, of which 1 million were of the 21–22 nt siRNA size class derived from the plus (+) strand of the Y-Sat RNA genome. From this (+) strand-specific pool, siRNAs corresponding to the yellow domain region form part of a siRNA hot spot along the Y-Sat genome (Figure S2). Furthermore, the 21–22-nt siRNAs starting at nt. 178, 180 and 181 accumulated at relatively high frequencies, with 1576, 2368 and 3352 reads detected respectively (Figure 2). The above results correlate with the proposal that the yellowing symptoms associated with CMV Y-Sat infection of tobacco are due to Y-Sat yellow domain-specific siRNA-directed silencing of the CHLI gene.

### Transformation of *N. tabacum* with a silencing-resistant version of CHLI prevents Y-Sat symptoms

**Figure 3:**
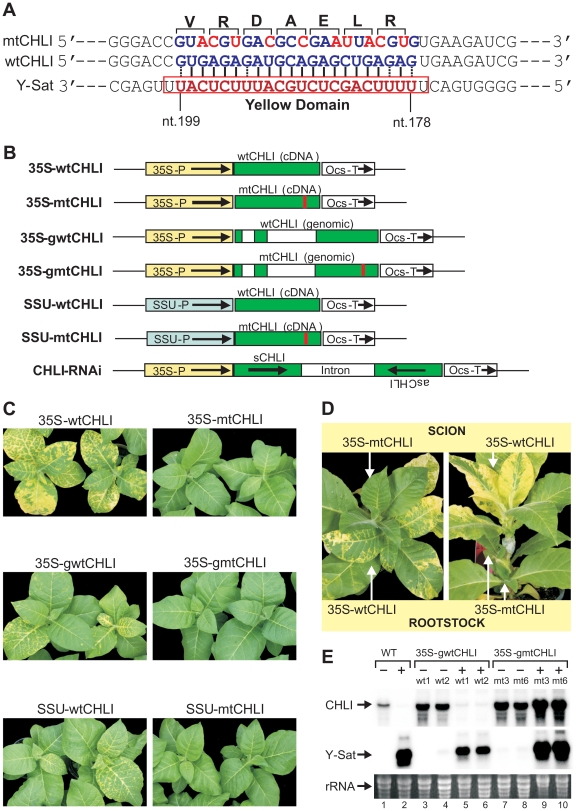
Transformation of *N. tabacum* with silencing-resistant CHLI constructs inhibits Y-Sat-induced yellowing symptoms. (***A***), Ten translationally silent nucleotide changes (shown in red) were introduced to the CHLI sequence to disrupt the binding between Y-Sat siRNAs and the CHLI mRNA. The amino acid sequence is given above. (***B***), Schematic diagrams of wild-type (wt) and mutant (mt) CHLI overexpression constructs plus the RNAi construct used for tobacco transformation. 35S-P, cauliflower mosaic virus 35S promoter; SSU-P, tobacco rubisco small subunit promoter; Ocs-T, *Agrobacterium tumefaciens* octopine synthase 3′ terminator region. Empty boxes represent introns of the CHLI genomic sequence; red lines indicate the sequence-modified region. The RNAi construct contains a partial (571 bp) sense (sCHLI) and antisense (asCHLI) CHLI sequence spanning the Y-Sat target site. (***C***), Tobacco plants transformed with wild-type CHLI constructs develop the yellowing symptoms upon CMV Y-Sat infection, but those transformed with mutant CHLI constructs do not show the symptoms. The picture was taken 20 days post-inoculation. Some of the CMV Y-Sat-infected wtCHLI plants showed a delayed onset of the yellowing symptoms, presumably because the CHLI transgene provided additional CHLI mRNA to the endogenous transcript, causing a reduction in the rate of CHLI silencing. (***D***), An example of reciprocal grafting between CMV Y-Sat-infected mtCHLI plants and wtCHLI plants. Only the wtCHLI scion or root stock developed the yellowing symptoms. (**E**), Northern blot hybridization shows that the CHLI mRNA expressed from the mtCHLI construct is resistant to Y-Sat-induced silencing. The ‘+’ symbol indicates samples from plants infected with CMV Y-Sat, while the ‘−’ symbol identifies samples extracted from plants prior to CMV Y-Sat infection. wt1, wt2, mt3 and mt6 are line numbers for the 35S-gwtCHLI and 35S-gmtCHLI transformants. Gel lane numbers are given at the bottom.

To determine if CHLI silencing alone was responsible for the Y-Sat-induced symptoms, we introduced 10-nt silent mutations into the 22-nt complementary sequence of the wild-type CHLI gene (wtCHLI), and transformed tobacco plants with the mutated version of the CHLI gene (mtCHLI). The modified CHLI transgene contains 10 nucleotide changes within the 22-nt region complementary to Y-Sat yellow domain siRNAs, bringing nine mismatches to this region of complementarity, rending it resistant to cleavage by these Y-Sat siRNAs (Figure 3*A*–*B*). Strikingly, the Y-Sat-induced symptoms were completely abolished in tobacco plants transformed with the mtCHLI constructs; none of the 44 independent transgenic lines developed yellowing symptoms upon CMV Y-Sat infection (Figure 3*C*; Table S1). This is in direct contrast to the population of tobacco plants transformed with the wtCHLI constructs, where 34 of 36 plant lines analysed showed yellowing symptoms upon CMV Y-Sat infection. The absence of symptoms in mtCHLI lines was not due to a lack of Y-Sat accumulation; mtCHLI plants grafted with diseased wtCHLI lines, either as the scion or rootstock, remained symptom free (Figure 3*D*). Northern blot hybridization analysis confirmed that the Y-Sat RNA accumulated to high levels in both wtCHLI and mtCHLI plants (Figure 3*E*). The CHLI transcript level was dramatically reduced in infected wtCHLI plants, but remained high in infected mtCHLI plants (Figure 3*E*, compare lanes 5–6 with lanes 9–10). These analyses indicated that the modified CHLI transcript was resistant to Y-Sat siRNA-directed silencing, allowing for sufficient accumulation of this modified transcript in infected plants for normal chlorophyll biosynthesis. These results also suggest that sRNA-directed silencing of CHLI alone is sufficient for the induction of the disease symptoms associated with CMV Y-Sat infection. Taken together, our findings strongly indicate that sRNA-mediated viral disease symptoms can be prevented through the introduction of a silencing-resistant version of a sRNA targeted host gene(s).

### Y-Sat disease-resistant *Nicotiana* species contain single-nucleotide variation in the siRNA-targeted CHLI sequence

**Figure 4:**
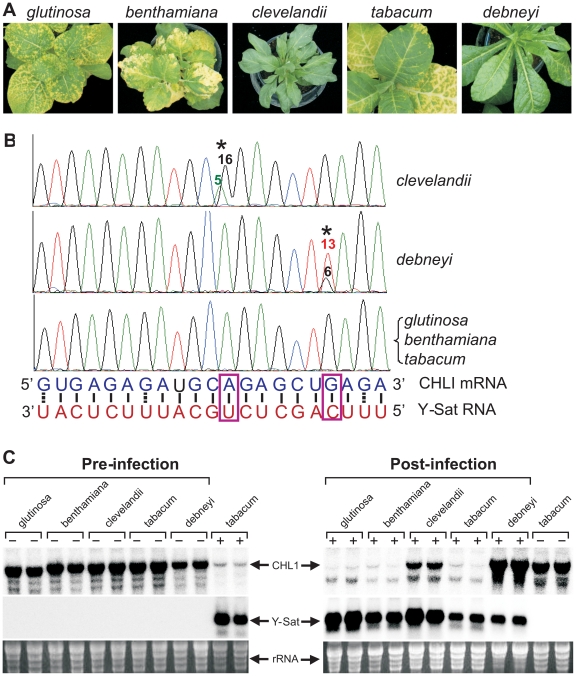
Natural sequence variation in the Y-Sat siRNA-targeted region prevents sRNA-directed silencing of CHLI and hence development of yellowing symptoms upon CMV Y-Sat infection. (***A***), Typical phenotypes upon CMV Y-Sat infection of three disease susceptible, *N. glutinosa*, *N. benthamiana* and *N. tabacum*, and two disease-resistant species, *N. clevelandii* and *N. debneyi*. (***B***), Sequencing trace files showing the presence of single nucleotide changes in the CHLI mRNA from the two disease-resistant species. The asterisk (*) indicates the variant nucleotide position, with the number of individual RT-PCR clones containing the specific nucleotide variant given above the corresponding trace file peak. (***C***), CHLI mRNA from *N. clevelandii* and *N. debneyi* is resistant to Y-Sat-induced silencing. The ‘+’ and ‘-’ symbols indicate samples from plants infected (+) or uninfected (−) with CMV Y-Sat.

Not all *Nicotiana* species are susceptible to Y-Sat-induced yellowing symptoms [15]. This suggests that sequence variations might exist in the coding sequence of the CHLI gene in some *Nicotiana* species, rendering these species ‘resistant’ or free of Y-Sat siRNA-induced CHLI silencing. We sequenced the CHLI transcript from five different *Nicotiana* species, including three disease-susceptible (*N. tabacum*, *N. glutinosa* and *N. benthamiana*) and two resistant (*N. clevelandii* and *N. debneyi*) species (Figure 4*A*). The three susceptible species which develop yellowing symptoms upon CMV Y-Sat infection had identical target sequences in the CHLI gene (Figure 4*B*). In contrast, of the two disease-resistant species which do not develop yellowing symptoms upon infection, *N. clevelandii* expressed two CHLI mRNA species (presumably because it is an allotetraploid containing two different chromosome pairs), with the predominant species containing an A to G change at the targeted CHLI sequence, converting tobacco's A:U base pairing to a weaker G:U wobble pair near the mRNA cleavage site (Figure 4*B*). Similarly, *N. debneyi* expressed two CHLI mRNA species with the predominant form containing a G to U conversion compared with the tobacco sequence, changing a strong G:C pairing to a U-C mismatch. Northern blot hybridization of CMV Y-Sat-infected and uninfected plants of these five *Nicotiana* species showed that CHLI expression was strongly silenced in infected plants of the susceptible species (Figure 4*C*). In contrast, CHLI mRNA levels remained high in infected *N. clevelandii* and *N. debneyi* plants suggesting that the mRNA variants expressed by these two symptomless species are resistant to Y-Sat siRNA-induced silencing. These results suggest that the previously observed host species specificity of satellite RNA-induced diseases [1], [2] results from sequence variation within the viral sRNA-targeted host gene(s). In addition, these results further demonstrate that the disease symptoms associated with CMV Y-Sat infection are solely due to silencing of the CHLI gene.

## Discussion

Our results demonstrate that the yellowing symptoms associated with CMV Y-Sat infection in tobacco result from Y-Sat siRNA-directed silencing of the chlorophyll biosynthetic gene CHLI. This finding is consistent with results from previous site-directed mutagenesis studies showing that Y-Sat mutants with nucleotide changes inside the yellow domain either retain or lose the ability to induce yellowing symptoms [13], [14]. In agreement with siRNA-directed silencing of CHLI solely accounting for the yellowing symptoms associated with CMV Y-Sat infection, Y-Sat variants which are capable of inducing such symptoms have higher degrees of sequence complementarity to the CHLI target sequence than those that can no longer induce yellowing upon CMV Y-Sat infection (13,14; Figure S3).

As observed for the yellowing symptoms associated with CMV Y-Sat infection, several other disease symptoms induced by viral satellite RNAs have also been associated with a short sequence within the respective satellite RNA genomes. For instance, the chlorotic phenotypes induced by the B2 and WL3 satellite RNAs of CMV in tobacco and tomato are determined by a ∼26-nt region (nt. 141–166) of the satellite genome [2], [5], [6]. Interestingly, BLAST searching with this sequence identifies a 21-nt match to a tobacco cDNA (accession # U62485) encoding a glycolate oxidase, which is involved in plant photorespiration and, when the expression of the gene is down-regulated, plants develop yellowing [18]. Furthermore, the necrotic symptoms induced by CMV Y-Sat infection in tomato are also associated with a short sequence, from nt. 309 to 334, of the Y-Sat genome [4]. Single nucleotide changes to these short sequences have been shown to dramatically affect both the severity and host specificity of these satellite RNA-induced disease symptoms [4]–[6]. Taken together, these observations suggest that siRNA-directed host gene silencing is a common mechanism for satellite RNA-induced symptoms. This mechanism could also be extended to viroids, another group of small non-protein-coding RNA pathogens in plants [19]. The pathogenicity of *Potato spindle tuber viroid* (PSTVd), a 359-nt non-protein-coding RNA pathogen, has been associated with two ∼20-nt partially complementary regions of the PSTVd RNA genome known as virulence-modulating regions [20]. Furthermore, expression of an hairpin RNA transgene derived from PSTVd in tomato resulted in the expression of symptoms similar to PSTVd infection [10], suggesting that a sRNA-directed RNA silencing mechanism is also responsible for the disease symptoms induced by PSTVd infection.

The siRNA-mediated disease mechanism reported here is consistent with the previous observation that disease induction by satellite RNAs involves the interaction of satellite RNAs, their helper viruses, and the host plant [1], [2]. Diseases caused by satellite-derived siRNA-mediated host gene silencing would require i) the existence of sufficient sequence complementarity between the satellite RNA genome and a host gene mRNA; ii) a helper virus that supports high levels of replication of the satellite; and iii) host RNA silencing machineries for efficient siRNA biogenesis and siRNA-directed mRNA degradation. One of the key helper virus-encoded factors for satellite-induced disease symptoms would be a RNA silencing suppressor protein. Most plant viruses encode silencing suppressor proteins, which inhibit sRNA-mediated silencing in their host [21]. Silencing suppressors from helper viruses could affect satellite siRNA-mediated diseases in two ways. The suppressor protein could inhibit host antiviral silencing to enhance the accumulation of satellite RNAs, or, it could act to inhibit satellite siRNA-induced silencing of complementary host gene sequences to minimize the disease symptoms. Consistent with the latter possibility, transgenic expression of the potyvirus silencing suppressor P1/HC-Pro in tobacco almost completely abolished the yellowing symptoms induced by CMV Y-Sat infection [10]. Also, the chlorotic symptoms induced by the B2 and WL3 satellites of CMV in tobacco are associated specifically with RNA2 of subgroup II CMV: the symptoms are diminished when the satellites are replicated by subgroup I CMV [22]. A recent study demonstrated that subgroup I CMV encodes a stronger silencing suppressor protein (2b, encoded by RNA 2) than subgroup II CMV [23], raising the possibility that the lack of strong B2 and WL3 satellite-induced chlorosis in the presence of subgroup I CMV is due to CMV 2b-mediated suppression of satellite siRNA-induced host gene silencing. Thus, viral-encoded silencing suppressor proteins may have a dual function, helping the virus or subviral agent to evade sRNA-mediated antiviral defence by preventing the silencing of viral RNAs, and minimizing symptom severity by inhibiting the silencing of host genes.

RNA silencing has been suggested to be a driving force for the evolution of subviral RNAs including viral satellite RNAs [1], [10]. These RNA species tend to form stable secondary structures that are resistant to siRNA-mediated degradation [10], [24]. Also, satellite RNAs have little or no sequence homology with their helper virus genome [1], and this is presumably to avoid the silencing of the helper viruses by satellite-derived siRNAs. Our findings have further implications for the involvement of RNA silencing in the evolution of viral satellite RNAs in plants. Satellite RNAs with extensive sequence homologies to host genes would result in silencing of the targeted gene(s), affecting the viability of the host plant. This in turn, would affect the survival of both the helper virus and its satellite RNA. Consistent with this scenario, there has been no report showing extensive sequence homology between satellite RNAs and their host genome.

As discussed for satellites and viroids, infection of plants with both RNA and DNA viruses is also associated with the accumulation of virus-derived siRNAs [21]. Furthermore, more than 100 miRNAs have been identified from animal viruses [25]. Whether sRNA-directed host gene silencing plays a wider role in plant and animal viral disease remains to be investigated. Nevertheless, numerous plant and animal viral sRNAs have been shown to match host gene sequences and therefore have the potential to down-regulate their expression [26]–[30]. This raises the possibility that at least some virus-induced diseases are due to viral sRNA-directed silencing of host genes. Recent advances in high-throughput DNA and RNA sequencing technologies are expected to result in complete genomic sequences, not only for the infecting viruses, but also for their respective hosts, providing new opportunities to identify host genes that are potentially targeted by virus-derived sRNAs.

We have demonstrated here that transformation of tobacco with a silencing-resistant version of the CHLI gene completely prevented the yellowing symptoms associated with Y-Sat infection. This finding offers a potential new strategy for preventing viral siRNA-mediated diseases in plants and animals. However, viral replication has relatively high error rates and viruses often exist as quasispecies (mixtures of minor sequence variants) [31]. Thus, a modified host target gene protected against siRNAs of the original virus could potentially be silenced by siRNAs from a variant virus with an altered sequence. Introducing multiple nucleotide changes into the target sequence of the host gene, as demonstrated for the modified CHLI sequence harbouring 10 single nucleotide modifications, could minimize the recurrence of host gene silencing by siRNAs of variant viruses.

## Materials and Methods

### Plant growth, viral infection, RNA isolation and analysis, BLAST searching, and high-throughput small RNA sequencing

All *Nicotiana* species were grown in a 25°C glasshouse with natural light. Infection of tobacco plants with *Cucumber mosaic virus* plus Y-Sat, total RNA extraction and northern blot hybridization were performed as previously described [32]. Solexa sequencing of small RNAs from Y-Sat-infected tobacco was carried out at the Allan Wilson Centre Genome Service (New Zealand). BLAST searching to identify Y-Sat-targeted tobacco genes was performed as follows: 21-nt segments of the Y-Sat yellow domain sequence (e.g. nt.177–197, nt. 178–198, nt. 179–199) were used to BLAST search common tobacco sequences (taxid:4097) of the NCBI database “nucleotide collection (nr/nt)”. This search identified the *N. tobacum* CHLI cDNA (accessions: U67064 and AF014053) as the “best” match with the Y-Sat sequence.

### Plasmid construction

Full-length genomic and cDNA sequences of the CHLI gene were amplified from total DNA and RNA using the NEB Long Amp Taq kit and Qiagen One Step RT-PCR kit respectively according to the manufacturer's instructions. The CHLI forward primer (5′ ATCTGGTACCAAAATGGCTTCACTACTAGGAACTTCC 3′) and reverse primer (5′ TCTAGTCTAGAAGCTTAAAACAGCTTAGGCGAAAACCTC 3′) were used for both the PCR and RT-PCR reactions. PCR products were purified using a QIAquick PCR purification kit (Qiagen), cloned into the pGem-T Easy cloning vector (Promega) and sequenced. The full-length genomic and cDNA sequences (see Text S1) were digested with *Kpn*I/*Xba*I and cloned into the intermediate vector pBC (Strategene).

To create the modified CHLI sequence (mtCHLI and gmtCHLI), a 630 bp sequence containing the Y-Sat targeted 22-nt sequence was amplified as two halves; i) the 5′ half was amplified with forward (WT-F1, 5′ TGGCACAATCGACATTGAGAAAGC 3′) and reverse (MT-R1, 5′ ACGTAATTCGGCGTCACGTACGGTCCCCACTTGGGGATGC 3′) primers that spanned the 22-nt CHLI target site and allowed for the introduction of modified nucleotides, and ii) the 3′ segment was amplified with a forward primer (MT-F2, 5′ GTACGTGACGCCGAATTACGTGTGAAGATAGTTGAGGAAAGAG 3′) that also contained modified nucleotides spanning the 22-nt CHLI target sequence overlapping with MT-R1 and a reverse primer (WT-R2, 5′ AGCAGTTGGGAATGACAGTGGC3′). The two amplified products were joined together using overlapping PCR with Pfu polymerase (Promega) to generate a 630 bp fragment with a modified Y-satellite target sequence. A 223 bp fragment, containing the modified target sequence, was released by *Pst*I and *Eco*RI digestion and used to replace the *Pst*I-*Eco*RI fragment of the wild type sequence in the CHLI cDNA, giving rise to the modified sequence mtCHLI. Digestion of the mtCHLI sequence with *Pst*I and *Bam*HI released the modified region which was used to replace the corresponding region in the wild-type CHLI genomic sequence, giving rise to the modified genomic clone gmtCHLI.

The wild-type and mutated cDNA or genomic sequences were digested with *Kpn*I and *Xba*I and inserted between the 35S promoter and the Ocs terminator in pART7 [33], and the resulting expression cassettes were cloned into the binary vector pART27 [33] as a *Not*I fragment. Similarly, the wild-type and mutant CHLI cDNA clones were cloned into a binary vector with the rubisco small sub unit promoter.

To prepare the RNAi construct, CHLI cDNA was digested with *Pst*I and *Xba*I releasing a 571 bp fragment spanning the Y-Sat target site. This fragment was cloned into the Gateway-based hairpin RNA gene silencing vector Hellsgate 12 which incorporates a spliceable intron for improved silencing efficiency [34], [35].

### Tobacco transformation

All plant expression vectors were introduced into *Agrobacterium tumefaciens* strain LBA4404 by triparental mating in the presence of the helper plasmid pRK2013. *Agrobacterium*-mediated transformation of tobacco was carried out as described previously [36], using 50 mg/L kanamycin as the selective agent.

### 5′ RACE

Total RNA (2 µg) was ligated to a 24-nt RNA adaptor (5′ AACAGACGCGUGGUUACAGUCUUG 3′) using T4 RNA ligase (Promega) at room temperature for 2 hours in 50 mM HEPES pH 7.5, 0.1 mg/mL BSA, 8% glycerol, 2 units/µL RNasein RNase inhibitor (Promega) and 0.5 unit/µL T4 RNA ligase (Promega). The ligation was purified by phenol-chloroform extraction and ethanol precipitation. The purified product was reverse-transcribed using a CHLI-specific reverse primer (5′ AGCAGTTGGGAATGACAGTGGC 3′). Primary PCR was then performed using a forward primer matching the RNA adaptor (5′ AACAGACGCGTGGTTACAGTC 3′) and the CHLI reverse primer (as above). The RT-PCR product was then amplified using the same forward primer with a nested CHLI reverse primer (5′ ATATCTTCCGGAGTTACCTTATC 3′). The nested PCR product was separated on a 2% agarose gel, purified with the Ultra Clean-15 DNA purification kit (Mo Bio Laboratories), and ligated into the pGEM-T Easy vector (Promega) for sequencing.

### Chlorophyll assay

Approximately 0.5 grams fresh weight of plant material was ground into fine powder in liquid nitrogen, mixed with 15 mL methanol and filtered through filter paper. Chlorophyll a and chlorophyll b were measured in a spectrophotometer (Biochrom WPA light wave II) at a wavelength of 663 nm and 645 nm respectively. Chlorophyll concentration was measured as nmol per gram of fresh weight.

## Supporting Information

Figure S1 Infection of *N. tabacum* with the CMV helper virus alone does not induce silencing of the CHLI gene. Approximately 10 µg of total RNA from uninfected (lanes 1–2) and CMV-infected (lanes 3–4) were separated in formaldehyde-agarose gel, transferred to Hybdond-N membrane, and hybridized with ^32^P-labelled antisense RNA of the CMV coat protein (CMV-CP) sequence.
(TIF)

Figure S2 Distribution of plus (+) strand-specific siRNAs along the Y-Sat genome. A total of 1 million 21 to 22-nt (+) strand Y-Sat siRNAs were obtained from the total sRNA sequencing population of approximately 4 million sRNA reads. Each point represents the number of reads (the Y-axis), and the position of 5′ terminal nucleotide of each detected siRNA along the Y-Sat genome (the X-axis). The black line on the bottom represents the full-length Y-Sat genome, in which the “yellow domain” is drawn in orange. Note that the “yellow domain” corresponds to a siRNA hot spot.
(TIF)

Figure S3 Results from the mutagenesis studies by Jaegle *et al.* (*Journal of General Virology* 71: 1905–1912 [1990]; Ref. #13 in the main text) and Kuwata *et al*. (*Journal of General Virology* 72: 2385–2389 [1991]; Ref. #14 in the main text) are consistent with CHLI silencing being the cause of Y-Sat-induced yellowing symptoms. As shown by the sequence alignment, Y-Sat mutants capable of causing the yellowing symptoms (Y5, Y7) have a higher level of sequence complementarity with the CHLI target sequence than those (Y6, MY5, MY6) that do not induce the yellowing symptoms. For instance, the Y5 sequence has stronger complementarity with the CHLI sequence than the original Y-Sat sequence. Y7 contains an introduced C-A mismatch, but this is compensated by the substitution of a G:U wobble pair with a strong G:C pair. All three non-disease-causing mutants (Y6, MY5, MY6) have more mismatches, or G:U wobble pairs, with respect to the CHLI sequence, than the original Y-Sat sequence. Underlined letters are the modified nucleotides in the Y-Sat mutants. Perfectly matched nucleotides are shown in blue and mis-matched ones in red. Green letters indicate nucleotides that can form G:U wobble pairs with the CHLI sequence. The Y-Sat ‘yellow domain’ sequence is boxed. The CHLI sequence matching the original Y-Sat sequence is shown in red. The ‘+’ and ‘−’ symbols respectively indicate the presence and absence of Y-Sat yellowing symptoms upon infection.
(TIF)

Table S1 Phenotypes of independent wtCHLI and mtCHLI transgenic tobacco lines in response to Y-Sat infection.
(DOC)

Text S1 Sequences of the tobacco CHLI gene.
(DOC)
