# Supplementary figures and images for: Viral Small Interfering RNAs Target Host Genes to Mediate Disease Symptoms in Plants
Source: PLoS Pathog. 2011 May 5;7(5):e1002022. doi: 10.1371/journal.ppat.1002022 (PMC3088724; doi:10.1371/journal.ppat.1002022)

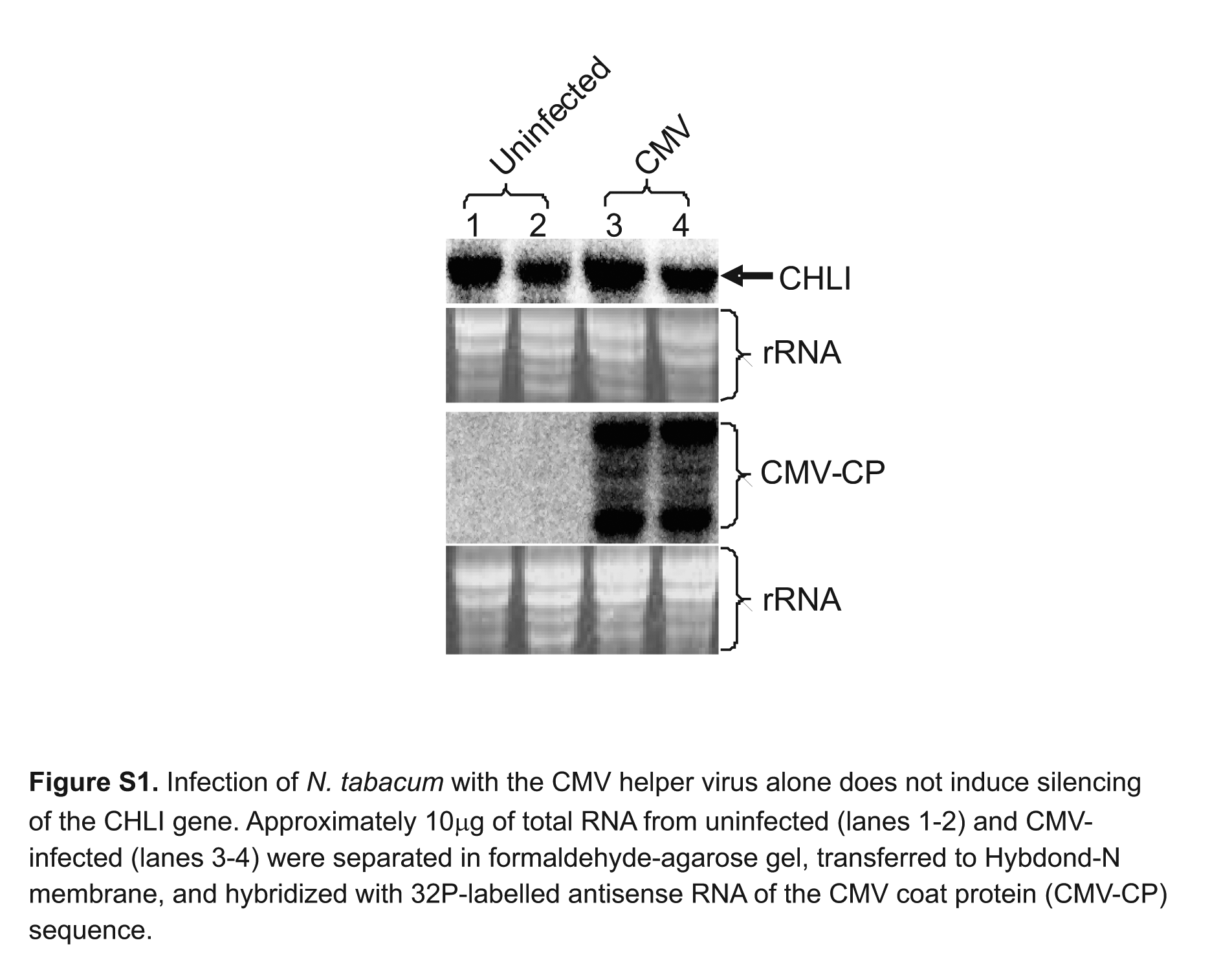

Supplement: Figure S1 — Infection of N. tabacum with the CMV helper virus alone does not induce silencing of the CHLI gene. Approximately 10 µg of total RNA from uninfected (lanes 1–2) and CMV-infected (lanes 3–4) were separated in formaldehyde-agarose gel, transferred to Hybdond-N membrane, and hybridized with 32P-labelled antisense RNA of the CMV coat protein (CMV-CP) sequence. (TIF) [file ppat.1002022.s001.tif]

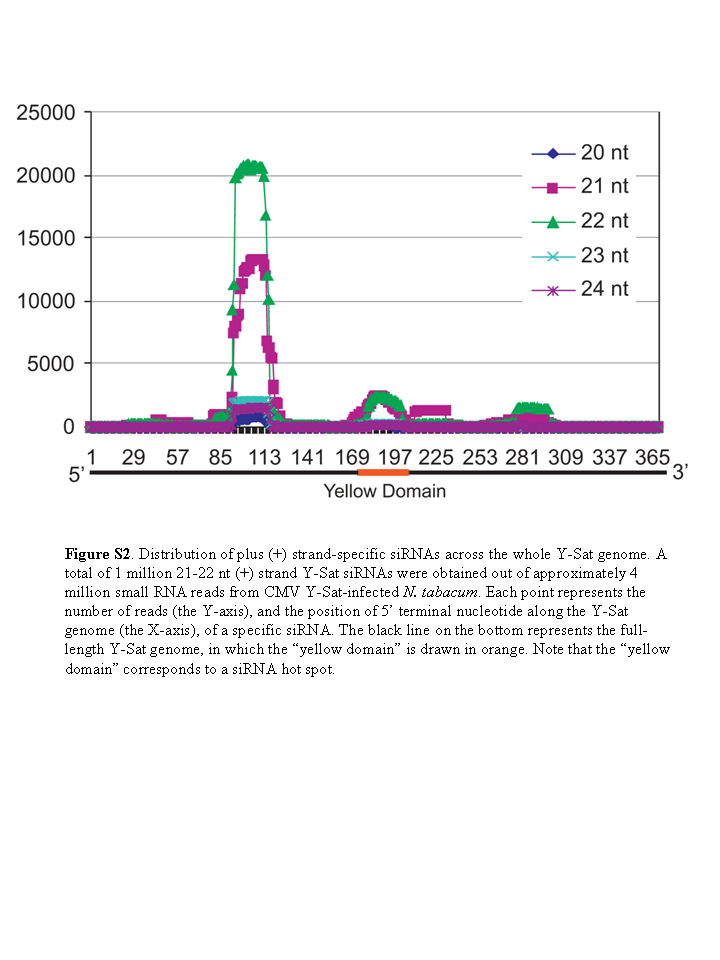

Supplement: Figure S2 — Distribution of plus (+) strand-specific siRNAs along the Y-Sat genome. A total of 1 million 21 to 22-nt (+) strand Y-Sat siRNAs were obtained from the total sRNA sequencing population of approximately 4 million sRNA reads. Each point represents the number of reads (the Y-axis), and the position of 5′ terminal nucleotide of each detected siRNA along the Y-Sat genome (the X-axis). The black line on the bottom represents the full-length Y-Sat genome, in which the “yellow domain” is drawn in orange. Note that the “yellow domain” corresponds to a siRNA hot spot. (TIF) [file ppat.1002022.s002.tif]

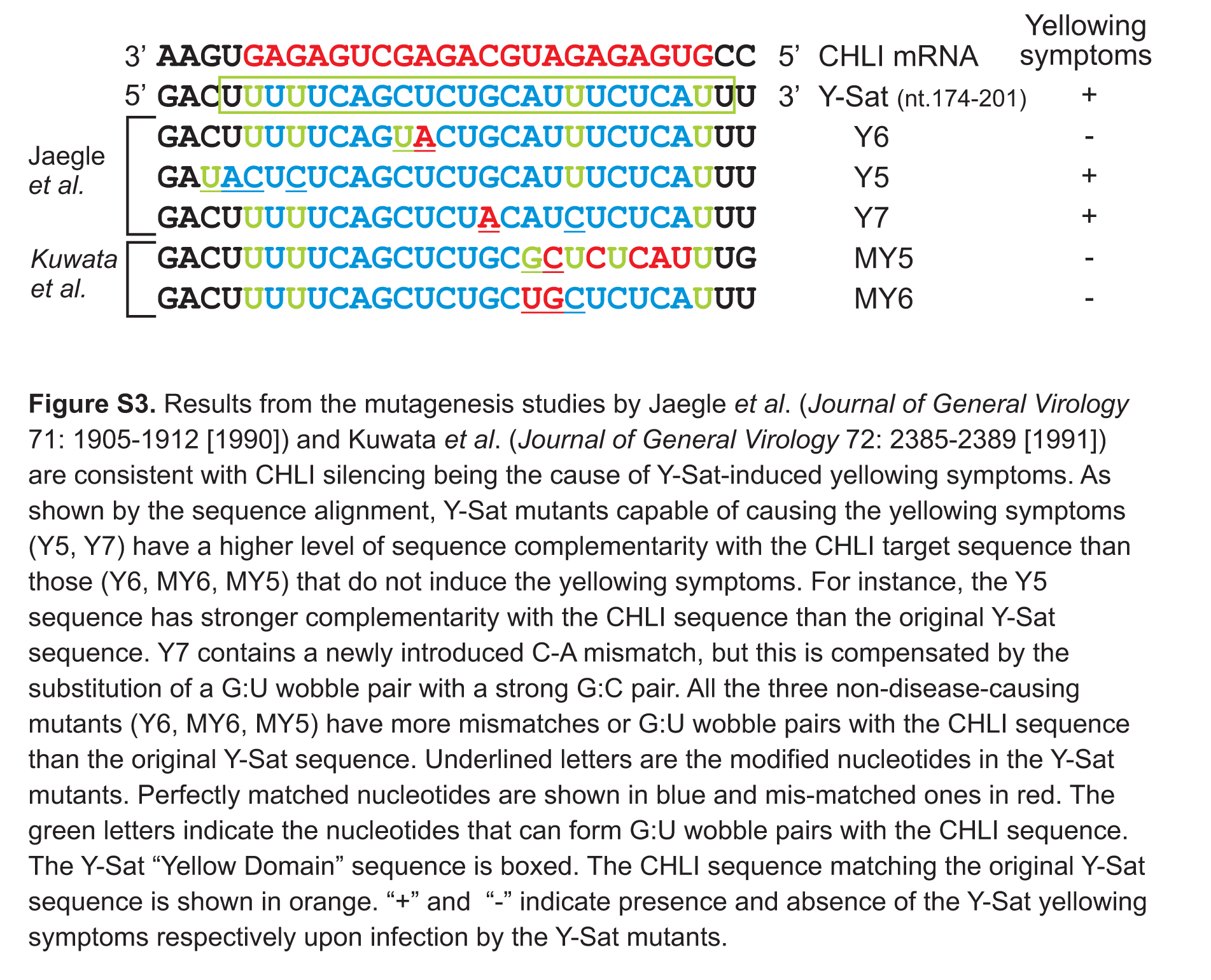

Supplement: Figure S3 — Results from the mutagenesis studies by Jaegle et al. (Journal of General Virology 71: 1905–1912 [1990]; Ref. #13 in the main text) and Kuwata et al. (Journal of General Virology 72: 2385–2389 [1991]; Ref. #14 in the main text) are consistent with CHLI silencing being the cause of Y-Sat-induced yellowing symptoms. As shown by the sequence alignment, Y-Sat mutants capable of causing the yellowing symptoms (Y5, Y7) have a higher level of sequence complementarity with the CHLI target sequence than those (Y6, MY5, MY6) that do not induce the yellowing symptoms. For instance, the Y5 sequence has stronger complementarity with the CHLI sequence than the original Y-Sat sequence. Y7 contains an introduced C-A mismatch, but this is compensated by the substitution of a G:U wobble pair with a strong G:C pair. All three non-disease-causing mutants (Y6, MY5, MY6) have more mismatches, or G:U wobble pairs, with respect to the CHLI sequence, than the original Y-Sat sequence. Underlined letters are the modified nucleotides in the Y-Sat mutants. Perfectly matched nucleotides are shown in blue and mis-matched ones in red. Green letters indicate nucleotides that can form G:U wobble pairs with the CHLI sequence. The Y-Sat ‘yellow domain’ sequence is boxed. The CHLI sequence matching the original Y-Sat sequence is shown in red. The ‘+’ and ‘−’ symbols respectively indicate the presence and absence of Y-Sat yellowing symptoms upon infection. (TIF) [file ppat.1002022.s003.tif]
