# Supplementary material for: Viral Small Interfering RNAs Target Host Genes to Mediate Disease Symptoms in Plants
Source: PLoS Pathog. 2011 May 5;7(5):e1002022. doi: 10.1371/journal.ppat.1002022 (PMC3088724; doi:10.1371/journal.ppat.1002022)
Supplement: Table S1 — Phenotypes of independent wtCHLI and mtCHLI transgenic tobacco lines in response to Y-Sat infection. (DOC) [file ppat.1002022.s004.doc]

**Table S1**. Phenotype of independent transgenic tobacco lines in response to Y-Sat infection

| Genotype | No. Independent lines infected | No. independent lines showing yellowing symptoms |
| --- | --- | --- |
| 35S-wtCHLI | 12 | 11 |
| 35S-mtCHLI | 20 | 0 |
| 35S-gwtCHLI | 12 | 11 |
| 35S-gmtCHLI | 12 | 0 |
| SSU-wtCHLI | 12 | 12 |
| SSU-mtCHLI | 12 | 0 |
| Non-transgenic tobacco | 6 | 6 |
