# Supplementary material for: Viral Small Interfering RNAs Target Host Genes to Mediate Disease Symptoms in Plants
Source: PLoS Pathog. 2011 May 5;7(5):e1002022. doi: 10.1371/journal.ppat.1002022 (PMC3088724; doi:10.1371/journal.ppat.1002022)
Supplement: Text S1 — Sequences of the tobacco CHLI gene. (DOC) [file ppat.1002022.s005.doc]

**Sequences of the tobacco CHLI gene**

*CHLI cDNA sequence cloned into pART7 (*Kpn*I/*Xba*I fragment)*

(Notes: Underlined letters indicate restriction sites used for cloning; bold letters in red shows the Y-Sat-targeted region; lower-case letters indicate intron sequences)

GGTACCAAAATGGCTTCACTACTAGGAACTTCCTCTTCAGCAGCAGCTGCAATATTAGCTTCTACAC

*Kpn*I

CCTTGTCTTCTCGCTCCTGTAAGCCTGCCGTTTTCTCCCTCTTCCCTTCTTCAGGGCAGAGTCAAGGGAGGAAGTTTTATGGAGGGATTAGAGTCCCAGTTAAGAAAGGGAGGTCCCAATTCCATGTGGCAATTTCAAATGTTGCGACGGAAATCAACCCTGCTCAAGAACAGGGTCAGAAACTTGCTGAGGAGAGCCAGAGACCGGTGTATCCATTTGCAGCTATAGTGGGACAAGATGAAATGAAGTTATGTCTTTTGCTGAATGTAATTGATCCAAAGATTGGAGGTGTGATGATAATGGGTGATAGAGGAACCGGGAAGTCCACCACGGTTAGATCTTTGGTAGATTTACTTCCTGAAATCAAAGTTATTTCTGGTGATCCGTTCAATTCAGATCCAGATGACCAAGAAGTAATGAGTGCAGAAGTCCGTGACAAATTGAGGAGCGGACAGCAGCTTCCTATATCTCGTACCAAAATCAACATGGTTGATTTACCGCTAGGTGCTACTGAGGACAGGGTGTGTGGCACAATCGACATTGAGAAAGCTCTTACTGAGGGTGTGAAGGCTTTCGAGCCTGGTCTTCTTGCTAAAGCTAACAGAGGAATACTTTATGTCGATGAGGTTAATCTTTTGGATGACCATTTAGTAGATGTTCTTTTGGATTCTGCAGCATCG

*Pst*I

GGATGGAACACTGTTGAAAGAGAGGGGATATCAATCTCACATCCGGCCCGATTTATCCTTATTGGTTCAGGTAATCCTGAAGAAGGAGAACTTAGGCCACAACTTCTTGATCGATTTGGAATGCATGCCCAAGTGGGGACC**GTGAGAGATGCAGAGCTGAGAG**TGAAGATCGTTGAGGAAAGAGCTCGTTTTGATAAGAACCCCAAGGAATTCCGTGAGTCATACAAGGCAGAGCAAGAAAAGCTCCAGAATCAAATCGACTCAGCTAGGAA

*Eco*RI

CGCTCTTTCTGCTGTTACAATCGATCATGATCTTCGAGTTAAAATCTCTAAGGTCTGTGCAGAACTAAATGTCGATGGATTGAGAGGTGATATAGTCACTAACAGGGCAGCACGAGCGTTGGCTGCACTAAAAGGAAGAGATAAGGTAACTCCGGAAGATATCGCCACTGTCATTCCCAACTGCTTAAGACACAGGCTGAGGAAGGATCCGTTGGAATCTATTGACTCGGGTGTACTTGTTGTTGAGAAATTTTATGAGGTTTTCGCCTAAG

*Bam*HI

CTGTTTTAAGCTTCTAGA

*Xba*I

*CHLI genomic sequence cloned into pART7 (*Kpn*I/*Xba*I fragment)*

GGTACCAAAATGGCTTCACTACTAGGAACTTCCTCTTCAGCAGCAGCTGCAATATTAGCTTCTACAC

*Kpn*I

CCTTGTCTCCTCGCTCCTGTAAGCCTGCCATTTTCTCCCTCTTCCCTTCTTCAGGtataaccaatcacaatgtagtttgcacaaattctatacgtacagttcaataattttaaagctaagttttcttgtactatgaatctgggttcttggaatttgatgggtactttgttattgcaggGCAGAGTCAAGGGAGGAAGTTTTATGGAGGGATTAGAGTCCCAGTTAAGAAAGGGAGGTCCCAATTCCATGTGGCAATTTCAAATGTTGCGACGGAAATCAACCCTGCTCAAGAACaggtactgtttttctacatttcggtattttgggatatggtaatttcattggggaagatttgtatataataaatagtgtgagatgttgggtttttggttgtggagcaacggtaaagttgtctctgtctaacctaggtcacgggttccagccgtgaaatggaacccgtgaaatcaaccattgatccttgcattagggtgggctgcctgcatcataccctttgggtgcagtcctctccggaccgtgtgtgaatacgagagtccgaaatctaagttgagggtatgaaatgaaatggcaatctaagttgaggatatgaaatgaaatggctgcaacattgcgagagagctagttaatttgttttctcgagtagctagaaagattattaaaatgcatataagttgtttgttgaaatgttcttggacccaattgtgatgcttcttggatgtgagtatatttcctatgtctcatattatatgagatgtttatttgacttgtacagtatattagcgatggtggaaatgaatattatactaatggttgaaaataagtttgatggaaaaaacatggcattgaaggttagaagttcggatattttaatgaattccaattcttgaaagttgtaaaatttctattgaaatggagtgatctcaaaaacatgtctggacgttcattttatgtgttttagaatagactctccgtttcattttatgtgttttagttggattgagcacgaggtttaaaaatgtaaagaagacttttgaatctcgcagtcttaatctaaatgtttgtataatgtaacaaagataacatttaaatcttgtggtctgaacttggcatgtcattcctataatgagtgtcattaagaaaaaaataggaatagtggaactaaagatttacagtactgaatatagaaagatggtagtatttttttaaacaaactaaaaaggaaaattaaaaacatatattgaaatggagggagtattaaatttgaggacaaggatgaatatataaagttcaaaatagtttattaggttcagattagaaagttctaacatttggggaaactggtttatttgcaaggcaatgtattctcaacccgtttttatttattgggttacgacacgaagtatatttataaactcacctactttgcgattttaAGGGTCAGAAACTTGCTGAGGAGAGCCAGAGACCGGTGTATCCATTTGCAGCTATAGTGGGACAAGATGAAATGAAGTTATGTCTTTTGCTGAATGTAATTGATCCAAAGATTGGAGGTGTGATGATAATGGGTGATAGAGGAACCGGGAAGTCCACCACGGTTAGATCTTTGGTAGATTTACTTCCTGAAATCAAAGTTATTTCTGGTGATCCGTTCAATTCAGATCCAGATGACCAAGAAGTAATGAGTGCAGAAGTCCGTGACAAATTGAGGAGCGGACAGCAGCTTCCTATATCTCGTACCAAAATCAACATGGTTGATTTACCGCTAGGTGCTACTGAGGACAGGGTGTGTGGCACAATCGACATTGAGAAAGCTCTTACTGAGGGCGTGAAGGCTTTCGAGCCTGGTCTTCTTGCTAAAGCTAACAGAGGAATACTTTATGTCGATGAGGTTAATCTTTTGGATGACCATTTAGTAGATGTTCTTTTGGATTCTGCAGCATCGGGATGGAACACT

*Pst*I

GTTGAAAGAGAGGGGATATCAATCTCACATCCGGCCCGATTTATCCTTATTGGTTCAGGTAATCCTGAAGAAGGAGAACTTAGGCCACAACTTCTTGATCGATTTGGAATGCATGCCCAAGTGGGGACC**GTGAGAGATGCAGAGCTGAGAG**TGAAGATCGTTGAGGAAAGAGCTCATTTTGATAAGAACCCCAAGGAATTCCGT

*Eco*RI

GAGTCATACAAGGCAGAGCAAGAAAAGCTCCAGAATCAAATCGACTCAGCTAGGAACGCTCTTTCTGCTGTTACAATCGATCATGATCTTCGAGTTAAAATCTCTAAGGTCTGTGCAGAACTAAATGTCGATGGATTGAGAGGTGATATAGTCACTAACAGGGCAGCACGAGCGTTGGCTGCACTAAAAGGAAGAGATAAGGTAACTCCGGAAGATATCGCCACTGTCATTCCCAACTGCTTAAGACACAGGCTGAGGAAGGATCCGTTGGA

*Bam*HI

ATCTATTGACTCGGGTGTACTTGTTGTTGAGAAATTTTATGAGGTTTTCGCCTAAGCTGTTTTAAGCTTCTAGA

*Xba*I
